# Cellular Network Power Allocation Algorithm Based on Deep Reinforcement Learning and Artificial Intelligence

**DOI:** 10.1155/2022/9456611

**Published:** 2022-06-24

**Authors:** Jinghua Cao, Xiang Zou, Rui Xie, Yujiang Li

**Affiliations:** ^1^Guangdong Songshan Polytechnic College, Shaoguan, Guangdong, China; ^2^Dalian Neusoft University of Information, Dalian, Liaoning, China; ^3^Guangdong University of Technology, Guangzhou, Guangdong, China; ^4^Lingnan Normal University, Zhanjiang, Guangdong, China

## Abstract

In the shortest path planning problem, the old algorithm usually has many defects, such as the robot's cognition being contrary to reality, the lack of practical operation feasibility, or the limitation of problem processing. Nowadays, with deep learning, artificial intelligence algorithms tend to be mature; it has become a mainstream trend to adopt end-to-end learning system instead of traditional old algorithms. In recent years, with the rise of the Internet of things emerging technology industry and the explosive surge of network data traffic, the drawback is the increasingly severe shortage of wireless spectrum resources. In order to effectively reduce the cochannel interference of D2D communication technology in the system and enhance the useable range of the cellular network, it is necessary to distribute the useful and efficient cellular resources of the system. In this article, we will study the D2D users and the selection scheme of D2D users' transmission power control mode and allocate the spectrum resources in the uplink of the cellular users in the communication network. In order to reduce the cochannel interference in a cellular network and improve the spectrum utilization of the system, the research direction of this article is to solve the problem of user communication resource allocation in a single-cell hybrid cellular network.

## 1. Introduction

In recent years, as freely movable robots have emerged in various fields and are widely used in factory production, military, aerospace, and daily life, how to make artificial intelligence robots operate in unknown and unfamiliar environments has become a hot issue. For example, in the field of warehouse logistics and transportation, classification robots that can move by themselves and gradually replace manual labor have entered the assembly line in the Amazon area [1]. The robots can automatically classify goods by region and store them in a specific area. In the military field, reconnaissance robots that can flexibly climb over obstacles for self-concealment and mine-clearance robots that automatically perform radar scanning have also been mass-produced and put into use in my country [2, 3]. The use of military robots directly reduces the risk of casualties. In the field of aerospace, “Yutu-2” has successfully landed on the lunar surface and conducted long-term exploration. In daily life, individual restaurants already have intelligent robots for food delivery and ordering, and even robots have penetrated into high-precision bank halls to answer questions and provide service consultations for customers. However, intelligent robots are not perfect [4]. There are still several difficult problems to be solved in the robot's travel: where they are, where their destination is, and how they travel is the optimal path. Since it is necessary to carefully and accurately determine the obstacles on the way, set up their own movement path to avoid potential risks, and achieve the highest effect in the shortest time, a suitable path calculation formula is often needed to ensure the accuracy and reliability of each movement.

## 2. Related Work

Mobile communication technology was born in the 1980s. After more than 40 years of continuous improvement and progress, it has realized the replacement from the first to the fourth generation of technology. It can be said that the speed is extremely amazing [5]. And there is official news that 5G technology will be fully implemented in 2020 and will be covered in a wide range. Mobile network data cannot be underestimated. It interconnects everything into a network and connects people all over the world into a system. The tree-like branches are intertwined into a network to build a huge information network. Literature elaborated on the far-reaching impact of the 5G era on people's production and life, especially on the country's economy. It has an irreplaceable and huge effect on my country's GDP and national economic development, and it also promotes my country's communications and information industries [6, 7]. The rapid development of high-tech industries. As of 2019, the number of mobile users worldwide has exceeded 9 billion. The mobile market report shows that the total amount of GSM/EDGE is showing a downward trend, and LTE users have exceeded the huge number of 2.5 billion. According to the data show that by 2023, users can reach as many as 5.5 billion people, its market share will exceed 60%, and it will gradually become the world's largest communications company giant [8]. At the same time, with the rapid development of the Internet industry and the Internet of Everything business, it has gradually developed into the communication and communication related to the Internet and intelligent robots. Some research predicts that the number of communication devices will increase by leaps and bounds by 2021 and will soon cross the 28 billion mark [9, 10]. The number of mobile terminals such as mobile phones, computers, and TVs that can be seen everywhere has also exploded in a positive correlation with time. In the era of big data, network communication and data traffic have also exploded. The two are closely related, and this growth is also a strict requirement for the current level of scientific research and application technology, such as faster speed, shorter latency, and greater throughput [11]. The future mobile communication technology will continue to develop and evolve in the direction of being higher, faster, more integrated, and more intelligent. The current literature has proposed a large number of solutions to the problems of resource shortage and low utilization efficiency. One is the home base station, which refers to small base stations with small coverage areas and concentrated scopes in different locations according to user distribution density, resource utilization, and allocation layout in a cell. The other is a typical representative of spectrum resource reuse technology-device-to-device direct communication technology, which allows communication devices within a short distance to establish direct communication with each other without the need for forwarding and assistance from the communication infrastructure. However, the spectrum resources used by these devices for communication need to be allocated reasonably, and an unreasonable allocation method cannot fully demonstrate the advantages of the D2D communication technology or even interfere with other communication users. The literature reported that in the future, 5G communication technology will penetrate every corner closely related to people's lives and will gradually establish a user-centric all-round wireless mobile communication ecosystem. Compared with 4G, the spectrum efficiency of 5G will increase several or ten times, and the energy efficiency and cost efficiency will even increase by more than a hundred times. Therefore, 5G can fully satisfy users' ultra-high-definition video, virtual reality, telemedicine, autonomous driving, and the strict network requirements of smart cities [12, 13].

## 3. Research on Deep Reinforcement Learning of Artificial Intelligence

In recent years, as mobile robots have become more and more popular in industries such as industry, military, aerospace, life, and entertainment, how to make robots move autonomously in unknown environments has become the most basic functional requirement [14–16]. For example, in terms of warehousing and logistics management, Amazon has realized the automated operation of mobile sorting robots, which can complete the function of positioning and sorting goods. In terms of national defense, our country has applied detection robots and mine-clearance robots that can surpass obstacles, which can effectively reduce casualties. In terms of aerospace, “Yutu-2” has successfully walked on the lunar surface for several days and nights. In the service industry, there are already mobile robots that provide food delivery in the catering industry and provide information consultation in the lobby of the bank. Usually, the path planning algorithm must be used to ensure that the optimal path is found. With the increasing complexity of actual application scenarios, mobile robots need to acquire the ability to learn independently to better adapt to the new environment. However, traditional mechanical path planning algorithms represented by algorithms often need to process environmental information first through image processing, which is very time-consuming and difficult to ensure the real-time performance of the algorithm. And when encountering a new unknown map, due to the lack of generalization and understanding of the nature of the problem, the mechanical algorithm will restart calculations. Therefore, it is very important to design an intelligent algorithm for self-learning path planning. Related research progress in the field of artificial intelligence makes it possible to realize an end-to-end system that maps inputs and outputs through models. Among them, the one that shines is deep learning, which relies on neural networks to fit functions and shows great advantages in computer vision and other fields. It can be used to easily extract image features for classification and further processing. At the same time, reinforcement learning can allow mobile robots to interactively obtain reward feedback during environmental exploration so as to learn and iterate the optimal decision sequence in a trial and error manner. Path planning is actually a series of multistep decision-making problems. The combined use of deep learning and reinforcement learning models will bring major changes to this field. Therefore, this topic introduces artificial intelligence algorithms into path planning for exploration, which has great significance and application value.

### 3.1. Research on Artificial Intelligence

Multistep decision-making is one of the core tasks in the research field of artificial intelligence. Intelligent agents perform a series of actions in an uncertain environment based on experience to achieve a set goal. Its potential applications will affect many fields, such as robotics, healthcare, smart grid, finance, and autonomous vehicles. The path planning problem studied in this article belongs to the category of multistep decision-making. The following will introduce the research status of deep learning and reinforcement learning, respectively.

#### 3.1.1. Current Status of Deep Learning Research

**Figure 1:**
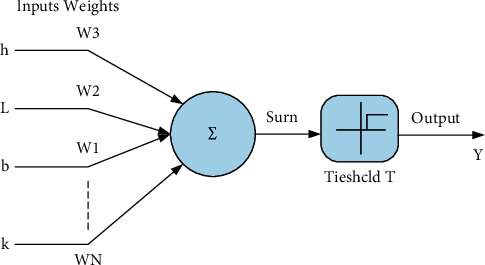
MCP mathematical model.

In 1943, neuroscientist McCulloch established a simple and abstract mathematical model imitating the structure of the biological nervous system; the MCP model is shown in Figure 1. Artificial neural networks began to try to simulate the thinking of the human brain. In 1958, Rosenblatt proposed perception and applied it to data classification. The perceptron can learn to update the weights through gradient descent to achieve convergence. However, then, the model was proved to be essentially a linear operation, and related research stalled.

**Figure 2:**
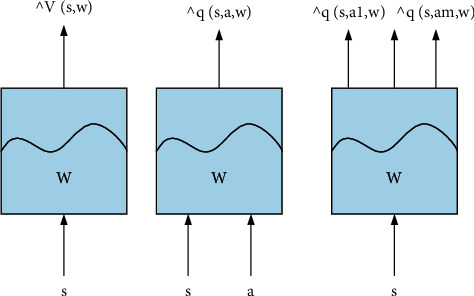
Approximate process of value function calculation by a neural network.

Neural networks are developing rapidly again, but due to the existence of the problem of gradient disappearance, the further improvement of deep learning encounters a bottleneck. The expressive ability of deep learning usually becomes stronger as the number of network layers increases. For this reason, in 2014, Simonyan proposed to further invent the extremely deep convolutional neural network VGG Net. Using a very small convolution kernel, the number of network layers can reach more than 16 layers. However, if the number of layers is further increased, the convergence will slow down, and the classification error will increase. Thus, Hekaiming et al. introduced the residual representation into the convolutional neural network and proposed ResNet. The results show that the method of learning residuals is more effective than the previous learning input-output mapping relationship, and the convergence speed is faster. The number of network layers can even reach 152 floors. One of the latest breakthroughs in deep learning is the capsule network proposed by Sabour et al. As shown in Figure 2, its core idea is to nest other neural layers in a single neural layer. It uses a protocol routing mechanism. When the neuron in the capsule processes an attribute of something, it will output the corresponding vector to all possible parent nodes. The nested neural layer vector output of each capsule adopts the Squashing function. Traditional convolutional neural networks cannot recognize the relative positions of objects, but CapsNet can learn different perspectives to improve accuracy due to the feature of homomorphism. Different from traditional *Q* learning, as shown in Figure 2, it uses neural networks to calculate Q values instead of states and actions. The main technology used is experience replay. The rewards obtained each time and the updated status will be saved for subsequent updates of the Q value. Since then, the combination of traditional reinforcement learning and deep reinforcement learning has kicked off.

**Figure 3:**
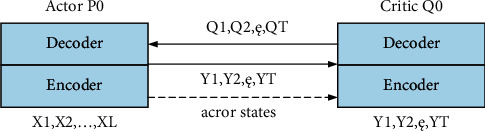
Encoding and decoding network relationship of actor evaluator algorithm.

Because the value-based learning algorithm represented by DQN has deficiencies in dealing with continuous actions, restricted states, random strategy problems, and other scenarios, strategy-based learning has begun to enter the field of research. By approximating the strategy as a continuous function, the optimal strategy can be found using the optimization of the strategy gradient. The representative one is the actor evaluator algorithm proposed by Benjio in 2017, which trains the neural network through the generated sequence. Among them, actors use strategy functions to interact with the environment and generate corresponding actions. The evaluator uses the value function to evaluate the performance of the actor and, at the same time, guide the actor's next action. In order to improve convergence, Timothy Plillicrap proposed a deep deterministic strategy gradient using dual networks and experience replay. At the same time, strong data correlation during experience playback will lead to poor training results. In order to solve this problem, Google's DeepMind team proposed the A3C algorithm. The algorithm sets up multiple threads, and each thread interacts with the environment separately and saves the results, which are then used to guide the learning interaction process, as shown in Figure 3.

**Figure 4:**
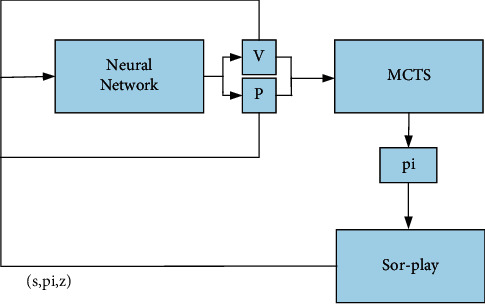
The combination of neural network and search tree.

After the hit AlphaGo defeated Li Shishi and Ke Jie, the world champions of go, DeepMind launched a more powerful AlphaZero. After a few hours of learning, they successively defeated go, chess, and Japanese Jiangqi programs. As shown in Figure 4, it skillfully combines the neural network and Monte Carlo search tree, uses the tree to optimize the parameters of the neural network, and uses the neural network to guide the book search. It is very effective in solving the problem type with a fully visible state and sufficient information.

### 3.2. Overview of Reinforcement Learning

**Figure 5:**
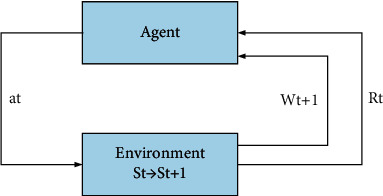
How to interact between agent and environment in reinforcement learning.

In reinforcement learning, the agent that makes the decision will observe the current state and select actions from the observation results. The environment will give reward feedback for the selected action, and the agent's status will be updated once. This is a loop until the agent reaches the termination condition. In this process, the interaction between the agent and the environment is shown in Figure 5.

In Figure 5, *f* represents the current cycle step, which represents the state, *r* represents the reward, and a represents the action. The general RL problem can be regarded as a discrete-time stochastic control process, as shown in the following equation. This cyclic process of reinforcement learning is usually represented by a Markov stochastic control process:

(1) $$\begin{array}{c} P\left(w_{i+1}|w_{1},a_{1}\right)=P\left(w_{i+1}|w_{1},a_{1},\ldots ,w_{0},a_{0}\right),\\ P\left(r_{i}|w_{1},a_{1}\right)=P\left(r_{i}|w_{1},a_{1},\ldots ,w_{0},a_{0}\right). \end{array}$$

The strategy in reinforcement learning defines how the agent chooses actions. Strategies can be classified according to whether they are static or not. The nonstationary strategy depends on the time step size and can be used in a limited time boundary. The cumulative reward that the agent seeks is limited to a limited number of steps in the future. Strategies can also be classified as deterministic or random: strategies can be described as follows:

(2) $$\begin{array}{c} V^{x}\left(s\right)=E\left[\sum_{k=0}^{\infty }\gamma^{k}r_{i+k}|s_{t}=s_{i}\pi \right],\\ r_{i}=\underset{a-x\left(x_{1}\right)}{E}R\left(s_{i},a_{i},s_{i+1}\right),\\ P\left(s_{i+1}|s_{i},a_{i}\right)=T\left(s_{i},a_{i},s_{i+1}\right),a_{i}-\pi \left(s_{it}\right). \end{array}$$

According to the definition of the expected return function, the best expected return can be defined as follows:

(3) $$\begin{array}{c} V*\left(s\right)=\underset{\pi \in \Pi }{\max}V^{\pi }\left(s\right). \end{array}$$

In addition to the *V* value function, the Q value function can also achieve the goal, which is defined as follows:

(4) $$\begin{array}{c} Q^{\pi }\left(s,a\right)=E\left[\sum_{k=0}^{\infty }\gamma^{k}r_{i+k}|s_{i}=s,a_{i}=a,\pi \right]. \end{array}$$

For MDP, the Bellman equation can be used to recursively rewrite formula (4):

(5) $$\begin{array}{c} Q^{\pi }\left(s,a\right)=\sum_{s^{\prime }\in s}T\left(s,a,s^{\prime }\right)\left(R\left(s,a,s^{\prime }\right)+\gamma Q^{\pi }\left(s^{\prime },a=\pi \left(s^{\prime }\right)\right)\right). \end{array}$$

Similar to the *V* value function, the optimal function of the Q value function can be defined as follows:

(6) $$\begin{array}{c} Q^{*}\left(s,a\right)=\underset{x\in \Pi }{\max}Q^{\pi }\left(s,a\right) \end{array}$$

Compared with the *V* value function, the special feature of the Q value function is that it can directly obtain the optimal function:

(7) $$\begin{array}{c} \pi *\left(s\right)=\underset{a\in A}{\arg\max}Q*\left(s,a\right). \end{array}$$

The optimal value function F' is the expected discount reward that the agent can obtain after adopting a strategy in a given state *S*. The optimal Q value function is the agent taking actions in a given state, and then following the strategy, the expected discount returns can be obtained. The difference between the Q value function and the *V* value function can also be used as the advantage function:

(8) $$\begin{array}{c} A^{\pi }\left(s,a\right)=Q^{\pi }\left(s,a\right)-V^{\pi }\left(s\right). \end{array}$$

A reinforcement learning agent usually consists of the following parts: it can predict the value function of each state or each state or action, strategy, or an environment model combined with the planning algorithm, including the estimated transfer function and reward function.

### 3.3. Overview of Deep Learning

Deep learning input to output function is as follows:

(9) $$\begin{array}{c} y=f\left(x,\theta \right). \end{array}$$

A deep neural network consists of multiple layers of neurons, and each layer contains a nonlinear transformation. These transformations allow different neural layers to learn different levels of abstract features. For example, for a neural network with only one fully connected hidden layer, the input layer is a feature column vector of size. The value of the next hidden layer is obtained by the nonlinear function transformation of the input vector.

(10) $$\begin{array}{c} h=A\left(W_{1}\cdot x+b_{1}\right), \end{array}$$

where *X* is the activation function and this activation function makes each layer conversion become nonlinear, thus providing the expression ability of the neural network. Under these circumstances,

(11) $$\begin{array}{c} y=\left(W_{2}\cdot x+b_{2}\right). \end{array}$$

**Figure 6:**
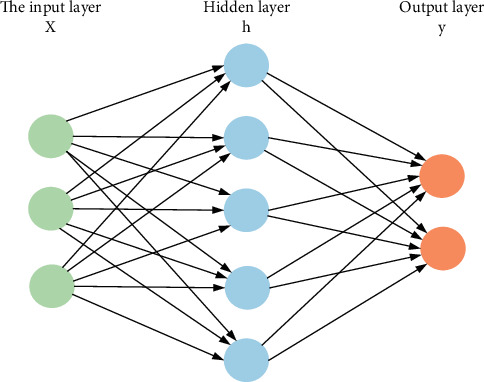
Neural network model.

The neural network model is shown in Figure 6.

The neural network layer is trained to minimize empirical errors. The optimization of neural network parameters usually uses the gradient descent method based on the back propagation algorithm. During each iteration, the internal parameters of the neural network will be adjusted to a smaller error:

(12) $$\begin{array}{c} \theta \leftarrow \theta -a\nabla_{\theta }I_{s}\left[f\right], \end{array}$$

where a is the learning rate. In addition to this simple feedforward neural network layer, other types of tragic networks have also been designed. Each kind of deformable neural network has its own application scenarios, and its advantages are also different. In supervised learning, the problems of bias and overfitting are weighed to different degrees. In addition, the number of layers of the neural network can also be changed arbitrarily. Under the recent trend, the number of layers has been increasing. In 2017, Szegedy and others have proposed an ultradeep neural network with more than 100 layers.

## 4. D2D Communication Mode Selection and Channel Allocation Plan

With the continuous development of wireless communication technology, the shortage of spectrum resources has become increasingly acute. D2D communication technology has become a key solution for spectrum reuse in contemporary mobile communication technology. This section proposes a joint solution for D2D communication mode selection and channel allocation, which is mainly for single-cell cellular networks. The algorithm implementation of this scheme is based on the maximum signal-to-noise ratio as the selection criterion, avoiding the introduction of serious cochannel interference and then using the greedy strategy to complete the mode selection and channel allocation of D2D communication users in a single-cell hybrid cellular network.

### 4.1. Scene Description

Based on the consideration of the stability of the system model involved, this article makes the following assumptions: the system model is an independent hybrid cell system including a central base station. There are two different communication users in this cell: cellular user CUE and D2D communication pair DUE, and CUE and DUE are evenly distributed in this cell. There are two different working modes for DUE, namely, multiplexing mode and dedicated mode. If multiplexing mode is selected, the uplink spectrum resources of the cellular system will be shared with the best CUE selected, and the DUE with dedicated mode will use idle cellular system spectrum resources. In order to ensure the communication quality of the CUE in the system, the transmission power of the CUE is maxCP, the maximum interference threshold is set to maxI, and to ensure the communication quality of the DUE, the maximum transmission power of DueT is PD, the minimum transmission power is DP, and the signal interference noise of DUE and the minimum value of the ratio is set to minDSINR. Assume that there are N cellular users in a cellular cell, represented by a set nC, and *M* D2D communication points, represented by a set mD (Yang et al., 2018). The number of users who choose D2D communication in the system is relatively small. In this article, it is assumed that the number of D2D communication pairs is less than the number of traditional cellular users.

### 4.2. D2D Communication Mode Selection and Channel Allocation Algorithm Based on Greedy Strategy

#### 4.2.1. Problem Description and Modeling

It can be seen that when the mth DUE reuses the nth CUE uplink spectrum resource, the mth DUE receiver DueR(m) will be interfered by the nth cellular user Cue(n). In order to ensure the normal communication of the mth DUE, the signal-to-interference and noise ratio of DueR(m) generally needs to meet the following conditions:

(13) $$\begin{array}{c} {\text{SINR}}^{D}\left(m\right)=\frac{P^{D}\left(m\right)Kd_{m}^{-\alpha }}{N_{0}+P_{\max}^{C}Kd_{m}^{-\alpha }}\geq {\text{SINR}}_{\min}^{D}. \end{array}$$

Among them, DPm is the transmit power in the mth DUE multiplexing mode, and md is the transmission distance between the mth DUE pair. In order to ensure the communication quality of the DUE, the minimum value of DueT(m) can be obtained from formula. The transmission power minDPm should meet the following:

(14) $$\begin{array}{c} P_{\min}^{D}\left(m\right)=\frac{{\text{SINR}}_{\min}^{D}\left(N_{0}+P_{\max}^{C}Kd_{m,n}^{-\alpha }\right)}{Kd_{m}^{-\alpha }}. \end{array}$$

Moreover, because DUE multiplexes CUE spectrum resources, DueT(m) will cause cochannel interference to CUEs sharing spectrum resources. In order to ensure the communication quality of CUE, the interference of DueT(m) to CUE cannot exceed a certain value. The cofrequency interference to the base station caused by the mth DUE multiplexing the nth CUE's spectrum resources must meet the following restrictions:

(15) $$\begin{array}{c} I_{n,m}^{C}=P^{D}\left(m\right)Kd_{m,ES}^{-\alpha }\leq I_{\max}. \end{array}$$

From the equation, it can be concluded that in the DUE multiplexing mode, the maximum transmission power maxDPm allowed by the transmitter DueT(m) is as follows:

(16) $$\begin{array}{c} P_{\max}^{D}\left(m\right)=\frac{I_{\max}^{C}}{K*d_{m,\text{ES}}^{-\alpha }}. \end{array}$$

Combining equations, in the multiplexing mode, the transmission power of DueT(m) should meet the following:

(17) $$\begin{array}{c} \max\left\{P_{\min}^{D}\left(m\right),\underline{P^{D}}\right\}\leq P^{D}\left(m\right)\leq \min\left\{P_{\max}^{D}\left(m\right),\bar{P^{D}}\right\}. \end{array}$$

When the DUE selects the dedicated mode, the DUE will select the CUE uplink communication in the idle state, which can be regarded as interference of the DUE to the base station. The interference of the CUE uplink is also zero, and the DUE allows the maximum transmission power PD to communicate to make it reach the maximum transmission rate. When the D2D communication pair shares the spectrum resources of the cellular user, the distance matrix between the DUE receiving end and the CUE device is as follows:

(18) $$\begin{array}{c} Dis^{C}=\left(\begin{array}{cccc} d_{1.1} & d_{1.2} & \cdots & d_{1.N}\\ d_{2.1} & d_{2.2} & \cdots & d_{2.N}\\ \vdots & \vdots & \ddots & \vdots \\ d_{M.1} & d_{M.2} & \cdots & d_{M.N} \end{array}\right). \end{array}$$

Among them, *N* represents the number of CUEs and *M* represents the number of DUEs. According to equations, the minimum transmission power matrix allowed by DUE in multiplexing mode is as follows:

(19) $$\begin{array}{c} P_{\min}^{D}=\left(\begin{array}{c} P_{\min}^{D}\left(1\right)\\ P_{\min}^{D}\left(2\right)\\ \vdots \\ P_{\min}^{D}\left(M\right) \end{array}\right)=\left(\begin{array}{cccc} P_{\min}^{D}\left(1,1\right) & P_{\min}^{D}\left(1,2\right) & \cdots & P_{\min}^{D}\left(1,N\right)\\ P_{\min}^{D}\left(2,1\right) & P_{\min}^{D}\left(2,2\right) & \cdots & P_{\min}^{D}\left(2,N\right)\\ \vdots & \vdots & \ddots & \vdots \\ P_{\min}^{D}\left(M,1\right) & P_{\min}^{D}\left(M,2\right) & \cdots & P_{\min}^{D}\left(M,N\right) \end{array}\right). \end{array}$$

The throughput of the system is an important criterion for measuring the cellular system. According to the above analysis, the system's mode selection and channel allocation optimization problem can be expressed as follows:

(20) $$\begin{array}{c} \underset{\mu_{m,n}}{\max}R_{\text{Total}}=\sum_{n=1}^{N}B\log_{2}\left(1+\frac{P^{C}\left(n\right)Kd{\left(n\right)}^{-\alpha }}{\mu_{m,n}I^{C}\left(n\right)+N_{0}}\right)+\sum_{m=1}^{M}B\log_{2}\left(1+\frac{P^{C}\left(m\right)Kd{\left(m\right)}^{-\alpha }}{\mu_{m,n}I^{D}\left(m\right)+N_{0}}\right),\\ \text{s.t.}:C1:P^{C}\left(n\right)\leq P_{\max}^{C},\;\forall n\in \left[1,N\right],\\ C2:P^{D}\leq P^{D}\left(m\right)\leq \underline{P^{D}},\;\forall m\in \left[1,M\right],\\ C3:\frac{P^{D}\left(m\right)Kd{\left(m\right)}^{-\alpha }}{I^{D}\left(m\right)+N_{0}}\geq {\text{SINR}}_{\min}^{D},\;\forall m\in \left[1,M\right],\\ C4:P^{D}\left(m\right)Kd_{m,\text{BS}}{\left(m\right)}^{-\alpha }\leq I_{\max},\;\forall m\in \left[1,M\right],\\ C5:\sum_{n=1}^{N}\mu_{m,n}\leq 1,\sum_{m=1}^{M}\mu_{m,n}\leq 1,\;\mu_{m,n}\in \left\{0,1\right\}. \end{array}$$

#### 4.2.2. Model Solving and Algorithm Design

**Figure 7:**
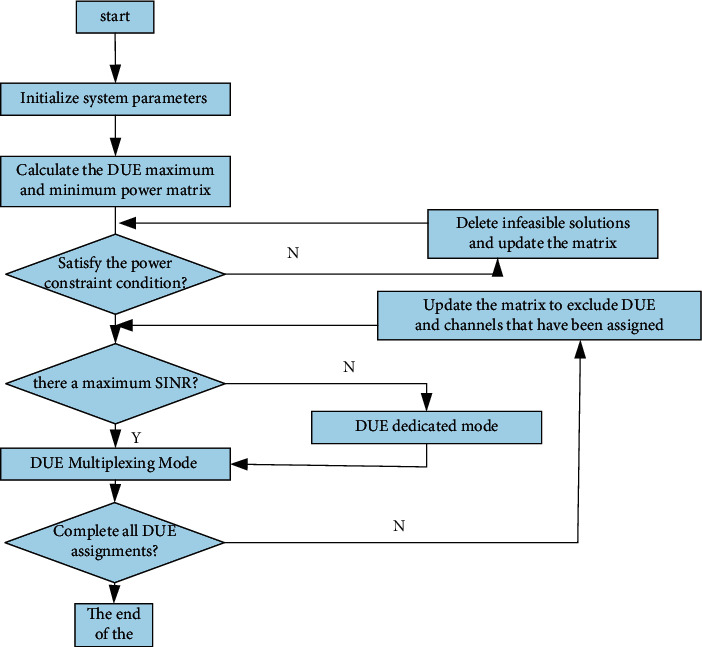
Algorithm flowchart.

Aiming at the optimization problem of mode selection and channel allocation in D2D communication in the single-cell hybrid cellular network, this section designs an algorithm for maximizing the total system throughput based on a greedy strategy. This algorithm excludes D2D communication pairs that cannot select multiplexing modes in relative positions, sets their working mode to the dedicated mode with the largest transmission power, thereby reducing the complexity of the algorithm in this article, and then selects the communication in the multiplexing mode in the system through the greedy strategy. The cellular uplink resource with the least interference is used as the communication link of the DUE. If there is a link with the least communication interference, it will be allocated to the DUE as a shared link resource. The D2D communication pair will select the multiplexing mode and allow it with power constraints. If it does not exist, the DUE selects the dedicated mode and works with the maximum power PD. The goal of this algorithm is to increase the access ratio of D2D communication users based on the maximum system throughput. The main steps are as shown in Algorithm 1 and Figure 7:

### 4.3. Experimental Simulation and Analysis

#### 4.3.1. Simulation Parameter Design

In this article, in a single cellular network scenario, the optimization problem solution proposed in this article is simulated and analyzed. It is assumed that several cellular users, several D2D communication pairs, and a central base station are evenly distributed in the cell. The algorithm uses MATLAB for simulation. The main system parameters are shown in Table 1.

#### 4.3.2. Analysis of Simulation Results

D2D users who choose the dedicated mode will work with the maximum allowable transmit power PD in the cellular network to maximize the total system throughput. The algorithm increases the proportion of D2D communication users who choose multiplexing modes while reducing the proportion of dedicated mode users. D2D users who choose the dedicated mode will work with the maximum allowable transmission power PD in the cellular network to maximize the total system throughput. The algorithm increases the proportion of D2D communication users who choose the multiplexing mode while reducing the proportion of users in the dedicated mode. Therefore, the total throughput of the communication users in the system will be smaller than the total throughput of the communication users in the system with a large proportion of users who choose the dedicated mode. The algorithm proposed in this article realizes that the throughput is maximized by reducing interference while taking into account the access ratio of the D2D multiplexing mode.

## 5. D2D Communication Power Control Scheme Based on Harmony Search

D2D communication technology provides a more convenient and flexible transmission mode for communication equipment. However, how to reasonably, effectively, and fully utilize traditional cellular spectrum resources and power allocation has become the focus of extensive research. The advantage of D2D communication technology is to improve the freedom of spectrum resource selection and spectrum utilization efficiency of communication users in the system, but coexisting with the traditional cellular communication method in the system will introduce cofrequency interference. Therefore, the harmony search algorithm can select the global optimal power and harmony combination so as to reasonably and effectively adjust the dynamic power of D2D communication users, avoid introducing serious cochannel interference, improve the communication service quality of users in the cellular network, and allocate them reasonably to system spectrum resources to maximize the total system throughput.

### 5.1. Model Establishment

As the DUE reuses the resources of the CUE, the CUE will be interfered with by the shared resource DUET, and the DUER will also be interfered with by the CUE. The interference of the mth DUE to the nth CUE can be expressed as follows:

(21) $$\begin{array}{c} I_{m,n}^{C}=P^{D}\left(m\right)Kd_{m,\text{BS}}^{-\alpha }. \end{array}$$

Among them, DPm represents the transmit power of the mth DueT. Similarly, it can be concluded that the receiving end of Due(m) is interfered with by Cue(n), which can be expressed as follows:

(22) $$\begin{array}{c} I_{m,n}^{D}=P^{C}\left(m\right)Kd_{m,n}^{-\alpha }. \end{array}$$

Among them, CPn represents the transmit power of the nth CUE. Assuming that in the system, when a Due(m) user multiplexes Cue(n) user uplink spectrum resources, the DSINRm of Due(m) is as follows:

(23) $$\begin{array}{c} {\text{SINR}}^{D}\left(m\right)=\frac{P^{D}\left(m\right)Kd_{m}^{-\alpha }}{I_{n,m}^{C}+N_{0}}. \end{array}$$

Among them, 0n is the white noise power spectral density and B is the channel bandwidth, which can be obtained by formula. According to Shannon's formula, the goal of power control in this article is to maximize the total rate of the cellular network system. The power control problem can be formally expressed as follows:

(24) $$\begin{array}{c} \underset{\mu_{m,n}}{\max}R_{\text{Total}}=\sum_{n=1}^{N}B\log_{2}\left(1+\frac{P^{C}\left(n\right)Kd{\left(n\right)}^{-\alpha }}{\mu_{m,n}I^{C}\left(n\right)+N_{0}}\right)+\sum_{m=1}^{M}B\log_{2}\left(1+\frac{P^{C}\left(m\right)Kd{\left(m\right)}^{-\alpha }}{\mu_{m,n}I^{D}\left(m\right)+N_{0}}\right)\text{,}\\ \text{s.t.}:C1:\underline{P^{D}}\leq P^{D}\left(m\right)\leq \bar{P^{D}},\;\forall m\in \left[1,M\right],\\ C2:\frac{P^{D}\left(m\right)Kd{\left(m\right)}^{-\alpha }}{I^{D}\left(m\right)+N_{0}}\geq {\text{SINR}}_{\min}^{D},\;\forall m\in \left[1,M\right],\\ C3:\frac{1}{m}\sum_{n=1}^{N}\sum_{m=1}^{M}\mu_{n,m}P^{D}\left(m\right)Kd{\left(m\right)}_{m,\text{BS}}{}^{-\alpha }\leq I_{\max},\;\forall n\in \left[1,N\right],\forall m\in \left[1,M\right],\\ C4:\sum_{n=1}^{N}\sum_{m=1}^{M}\mu_{n,m}P^{D}\left(m\right)\leq P^{\text{total}},\forall n\in \left[1,N\right],\;\forall m\in \left[1,M\right],\\ C5:\sum_{n=1}^{N}\mu_{m,n}\leq 1,\sum_{m=1}^{M}\mu_{m,n}\leq 1,\;\mu_{m,n}\in \left\{0,1\right\}. \end{array}$$

Among them, *N* represents the number of CUEs and *M* represents the number of DUEs.

### 5.2. Experimental Simulation and Analysis

#### 5.2.1. Simulation Parameter Design

This article simulates and analyzes the solution to the optimization problem proposed in this article in a single cellular network scenario. It is assumed that in a hybrid cell with a radius of 500 m, the base station is set up in the center of the communication network, and the cell is uniform. Several cellular users and D2D communication pairs are distributed. According to Table 2, design the main system parameters of the simulation scene and then use MATLAB software for algorithm simulation.

#### 5.2.2. Analysis of Simulation Results

The system simulation is realized by MATLAB programming, and the system parameters in Table 2 are used to complete the program design. This article focuses on the total throughput of system users and system communication users and the total interference of the same frequency to cellular users sharing resources in the multiplexing mode; a comparative analysis of algorithms is performed. Aiming at the problem of cochannel interference in the process of introducing D2D communication technology into cellular networks, a resource allocation scheme based on an adaptive global harmony search algorithm is proposed. Through the establishment and analysis of various constraints in the resource allocation process, a mathematical model of the resource allocation plan is constructed. According to the characteristics of the system with complex constraints, the algorithm proposes a problem solution based on an adaptive global harmony search algorithm and uses a multiobjective optimization method to obtain the optimal solution of the objective function. The experimental results show that the D2D communication power control scheme based on harmony search and the total throughput of the D2D communication user system are significantly better than the simulated annealing algorithm and the basic harmony search algorithm, and the scheme can effectively guarantee the communication service quality of each D2D communication user.

## 6. Conclusion

This article mainly studies how to use deep reinforcement learning, an end-to-end intelligent algorithm, to solve the path planning problem of a two-dimensional grid environment map. Through the training and learning of the deep reinforcement learning model, an agent can predict the shortest path of an unknown environment map, which has good generalization. However, based on the analysis of the existing reflective neural network and reinforcement learning model, it is found that they have some defects in the task of path planning, such as insufficient planning ability, accumulated error, and less use of key local information. Therefore, this article improves the structure of the neural network, value iteration module, and training process. In this project, the traditional cellular users and D2D communication users in a single-cell hybrid cellular network are taken as the research object, and the main purpose is how to alleviate the cochannel interference caused by the introduction of D2D communication technology through reasonable and effective radio resource allocation to the D2D communication users in the hybrid network, maximize the system throughput, ensure the quality of communication service, and ensure the fairness of users.

## Figures and Tables

**Algorithm 1:**
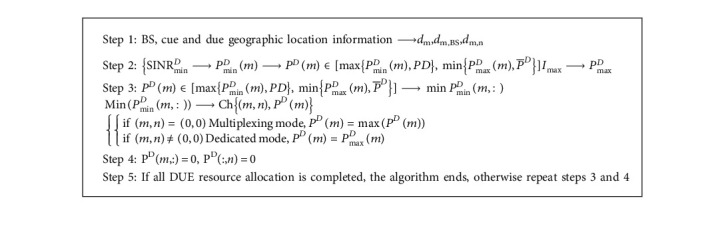
D2D communication mode selection and channel allocation.

**Table 1:** Main system parameters 1.

Parameter	Parameter value
Cell radius	500 m
Number of cellular users *N*	200
Subchannel bandwidth	180 KHz
D2D communication logarithm *M*	5, 10, 1520, 25
Minimum distance from user to ..l.the base station	25 m
D2D communication pair maximum distance	50 m
Maximum transmit power of cellular users	24 dBm
D2D user transmit power	[5, 24] dBm
Maximum interference value for cellular users	3 dBm
Minimum signal-to-interference noise ratio for D2D users	5(lBm
Noise power spectral density	−174 dBm/Hz
System path loss attenuation constant coefficient	4
Path loss attenuation index	0.8

**Table 2:** Main system parameters 2.

Parameter	Parameter value
Cell radius	500 m
Number of cellular users	200
Subchannel bandwidth	180 kHz
D2D communication logarithm	5, 10, 1520, 25
Minimum distance from user to the base station	25 m
D2D communication pair maximum distance	50 m
Maximum transmit power of cellular users	
D2D user maximum transmit power	24 dBm
D2D user minimum transmit power	5 dBm
Maximum interference value for cellular users	3 dBm
Minimum SINR for D2D users	5
Noise power	−174 dBm/Hz
Path loss attenuation factor	4
Path loss attenuation index	0.8

## Data Availability

The data used to support the findings of this study are available from the corresponding author upon request.
